# Mental health service use among migrant and Swedish-born children and youth: a register-based cohort study of 472,129 individuals in Stockholm

**DOI:** 10.1007/s00127-021-02145-2

**Published:** 2021-07-28

**Authors:** Ester Gubi, Hugo Sjöqvist, Karima Viksten-Assel, Sofie Bäärnhielm, Christina Dalman, Anna-Clara Hollander

**Affiliations:** 1 Department of Global Public Health, Karolinska Institutet, 171 77 Stockholm, Sweden; 2 Transcultural Centre, Region Stockholm, Stockholm, Sweden; 3 Center for Epidemiology and Community Medicine, Region Stockholm, Stockholm, Sweden

**Keywords:** Child and adolescent health, Migrants, Refugees, Health services utilization, Mental health and psychiatry

## Abstract

**Purpose:**

Migrant children underutilize mental health services (MHS), but differences according to age, reason for migration, type of problem, and time have not been thoroughly analyzed. We aimed to explore utilization of MHS among migrant children and youth and to study if the hypothesized lower utilization could be explained by fewer neurodevelopmental assessments.

**Methods:**

A cohort of the population aged 0–24 years in Stockholm, comprising 472,129 individuals were followed for maximum 10 years, between January 1, 2006 and December 31, 2015. We categorized individuals as accompanied refugee migrants, unaccompanied refugee migrants and non-refugee migrants, or Swedish-born. We used survival and logistic analyses to estimate rates of utilization of MHS.

**Results:**

Migrant children and youth utilized less MHS than the majority population, with hazard ratios ranging from 0.62 (95% CI: 0.57; 0.67) to 0.72 (95% CI: 0.69; 0.76). Refugee and non-refugee children utilized less mental health care than their Swedish peers, apart from the youngest refugees (0–10 years) who had similar utilization as Swedish-born. The lower rates were partly explained by all migrant youths’ lower risk of being diagnosed with a neurodevelopmental condition. Time in Sweden had a major impact, such that unaccompanied refugee minors had a higher utilization in their first 2 years in Sweden (OR: 3.39, 95% CI: 2.96; 3.85).

**Conclusion:**

Migrant youth use less MHS compared with native-born peers, and this is partly explained by fewer neurodevelopmental diagnoses. Strengthening the awareness about unmet needs, and the referring capacity by professionals in contact with migrant children could help reduce barriers to care.

**Supplementary Information:**

The online version contains supplementary material available at 10.1007/s00127-021-02145-2.

## Introduction

The number of migrants in the world is at an all-time high. Until 2015, Sweden had one of the most generous asylum policies in Europe, and currently, 20% of the population is foreign-born [1]. While most migration to Sweden historically has been labor-related, migration from outside of Europe has consisted mainly of asylum seekers—including many children and youth refugees, whose mental health needs have drawn increased attention. Less attention has been directed at the mental health needs of non-refugee migrant youths [2].

Despite extensive evidence that refugee children are at increased risk of poor mental health compared to their majority peers [3–5], there has only recently been intensified efforts to study *utilization* of mental health services among migrant and refugee children. Furthermore, studies investigating utilization have not consistently distinguished non-refugee from refugee sub-groups (for a recent exception, see Ref. [6]), notwithstanding the evident heterogeneity of migrant sub-populations. Given existing evidence that refugee children at increased risk of poor mental health, while studies on non-refugee children’s mental health are inconclusive [7], it is crucial to investigate mental health service use distinguishing between refugee and non-refugee groups to relate new results to current findings regarding needs.

### Indications of barriers to care

Previous studies show problematic trends in terms of utilization of mental health services among migrant children and youth within the Scandinavian universal health care system. While findings indicate an under-utilization of out-patient specialist psychiatric care among migrant children, emergency psychiatric and inpatient care have been found higher among refugee children compared to their majority peers [8–10], suggesting that refugee children access care at a higher severity of symptoms [11] and that migrant children overall face barriers to out-patient services. A systematic review of migrant children’s health care utilization confirmed this picture; while migrant children had reduced use of most types of health services, including mental health care, migrant children’s use of emergency and hospital services was higher than native populations’ use of such services [12].

In fact, research indicates that young people generally, and migrant youth in particular, face a wide range of barriers to mental health care. Certain barriers are prevalent regardless of migration background, such as a preference for self-reliance, perceived stigma, and problems recognizing symptoms [13], while others are specific to migrant and ethnic minority populations. For example, negative perceptions about the social consequences of mental illness for the individual and the family [14, 15], internalized and treatment stigma [16, 17], parental perceptions of lack of cultural competency among providers [18], cultural differences in explanatory models of illness, and cultural variations in knowledge and beliefs about mental health conditions [19, 20], have all been indicated as barriers to help-seeking and mental health service use. Moreover, low trust in the health care system, lack of awareness of services, linguistic obstacles [21], as well as acculturation gaps between generations may impede service use among migrant children and youth in need of care [22].

While help-seeking for mental health problems for children and adolescents are influenced by parental perceptions and behavior [23], other agents, such as schools [24] and child health care centers, likely play an important role in guiding and referring pre-school and school-aged migrants to mental health services. Thus, examining utilization among migrant children of all ages allows for a more complete understanding of service utilization, including the youngest age-groups whose utilization up to date has not been well examined [6, 9, 10]. Moreover, several studies show that migrant children are significantly less likely to be prescribed ADHD-medication or to be diagnosed with high functioning autism spectrum disorder (ASD) compared to majority individual [8, 25, 26]—despite a lack of evidence that the prevalence of such disorders are lower among migrants.. Therefore, a possible under-diagnosis of these conditions may be at play [27], although some studies suggest a higher risk of being diagnosed with ADHD among descendent children to immigrant parents, and an increased likelihood to be diagnosed as parental time in the new country increases [9, 28]. Nonetheless, given that disparities in diagnosis and treatment of neurodevelopmental disorders have been demonstrated among ethnic and migrant groups [29, 30], it is fruitful to investigate whether the under-utilization of psychiatric care among migrants that has been observed could in part be explained by the lower utilization of care related to neurodevelopmental conditions.

### Aims of this study

Using an outcome measure that includes visits to primary care, specialist psychiatric care, and specialist child and youth clinics, we aim to investigate the hypothesis that migrant children utilize less mental health services than Swedish-born, and that reason for migration, parental presence, time in Sweden, and country of origin explain some of the differences we expect to see. In addition, we aim to study if the hypothesized lower mental health service use among migrants is explained by fewer diagnoses of neurodevelopmental disorders.

## Methods

### Study population

The study population consisted of children and young adults (*n* = 591,816), born between 1991 and 2011 and residing in Stockholm county between January 1, 2006 and December 31, 2015 (ages 0–24 years). All study individuals had Swedish permanent residence permits and equal entitlements to the Swedish health care system. It was an open cohort with a minimum follow-up of 1 year. Individuals were censored at first occurrence of the outcome, leaving Stockholm, death, or at the end of study period. We included Swedish-born individuals with at least one Swedish-born parent and all individuals who were born abroad with two foreign-born parents. We excluded children born in Sweden to two migrant parents (*n* = 98,059), given that this group has been shown to differ in terms of mental health needs compared to majority individuals [31], and whose utilization was thus considered beyond the scope of this study. Individuals who did not reside in Stockholm during any of the study period (*n* = 4552), who were not registered as unaccompanied, but whose parents had discontinuous income data (*n* = 2773), had missing country of birth data (*n* = 2242), and adopted individuals (*n* = 5671) were excluded. Individuals who were born abroad with at least one Swedish-born parents (*n* = 6392) were also excluded, on the basis that having a Swedish-born parent could lower barriers to care (but they were included as a distinct category for sensitivity analyses). For the analyses which included unaccompanied minors (*n* = 1277), a total of 472,145 individuals were part of the final models; when we adjusted for parental socio-economic status, unaccompanied were not included, since such data were unavailable for this group.

### Data sources

Data were extracted from a linked register database called Psychiatry Sweden held by our research group, which consists of several national and regional registers. For the exposure data, this study included the total population register (for identification of individuals), the multi-generation register (for linking parents and children), the longitudinal integration database LISA (for income and education data), and the longitudinal database for studies of immigrants’ integration called STATIV (for migration data) (see Ref. [32] for more details on the registers). For the outcome data, the registers used were the prescribed drug registry (for psychotropic medication prescription) and the Stockholm county VAL-data-bases, with data on primary care, child and adolescent care, and specialized psychiatric care, including local registry-data from the child and adolescent psychiatric clinics in Stockholm (called the BUP/Pastill-database, see Ref. [33] for a detailed description of the different health care data registers).

### Exposure variables

The primary exposures in this study were migration background for the children and young adults, with the following main categories: Swedish-born children and young adults, defined as individuals born in Sweden with at least one Swedish-born parent (henceforth called Swedish) and migrant children and young adults, defined as foreign-born with two foreign-born parents (henceforth called migrants).

In the migrant sub-population, the children and young adults were categorized as refugee or non-refugee minors. Individuals were defined as refugees if parents had been granted residence permit as refugees by the Swedish board of Migration, based on the United Nations refugee convention. Some individuals lacked parental migration data, but were defined as refuges if they themselves had been registered as such. Among refugee children, there is a sub-group of unaccompanied refugee minors defined according to UNHCR [34] as children who have been separated from their parents, relatives, or other legal guardians. Individuals were defined as unaccompanied if they were registered as such in the STATIV database.

### Secondary exposures

To explore the effect of country of birth, we used data from Statistics Sweden, which groups country of birth into wider regions for confidentiality purposes. We used the following regions of origin for our descriptive statistics: Africa south of Sahara; Asia; Eastern Europe, Russia and the Baltics; Middle East and North Africa; Nordics; South America; Sweden; USA, Canada and Oceania; Western and Southern Europe. For our regression analysis, further grouping of regions was made.

### Outcome variables

The primary outcome was utilization of any mental health services, defined as first contact with primary care or specialist pediatric care (in Swedish, BUMM) for a psychiatric condition (ICD codes F-00-F99, X60-X84, Y10-Y34), first-time contact with specialized in- or out-patient psychiatric care (in Swedish, BUP), or first-time prescription of a psychotropic drug (antidepressants, ADHD-medication, sedatives, neuroleptics, or tranquilizers). First contact was defined as direct contact (excluding telephone contacts) with a doctor, psychologist, therapist, or mental health assistant nurse. The BUP/Pastill-database lacked information on type of caregiver and telephone contacts, and here, the variable “first visit” was used for first-time contact. The earliest date any of the above outcomes occurred was defined as the event.

The secondary outcome was first-time diagnosis with neurodevelopmental disorder, defined as ICD-10 F84 and F90, or prescription of ADHD-medication (ACT-code: N06B). The BUP/Pastill-database uses its own diagnostic system based on DSM, and here, diagnoses including pervasive developmental disorder and hyperactivity and attention-deficit syndromes were coded as equivalent to the ICD-10 codes above.

### Covariates

Sex and parental income were considered potential confounders and analyzed where possible. When unaccompanied migrants were included as a sub-group, data on parental income could not be included in the analysis. We used individual disposable family income from the LISA database. This variable is estimated by Statistics Sweden as an annual disposable income based on total family income from all sources (including welfare benefits, wages, pensions, etc.), while weighting the total household income with the number of children and their ages. The income variable was then categorized into quintiles based on the total population of each year. When an individual had parents with differing income values for the same year, the average of that year was used. The income variable was treated as a time-varying confounder, allowing for variation over each year of the study period.

Age was considered a potential confounder and categorized into 5-year categories in all regression models except for the time-in-Sweden analysis, where instead it was treated as continuous variable.

For migrants, time in Sweden was calculated based on an individual’s date of immigration and date of entry into the study.

### Statistical methods

We described basic characteristics for the study population according to migration background, age, sex, region of origin, and parental income at study start. We estimated hazard ratios and 95% confidence intervals for utilization of any mental health services according to our exposure categories, using a time-dependent survival analysis. In this model, age was used as underlying timescale, such that each individual’s time-to-event was based on age at entry to age at event or censoring. Using age as time scale allows for an analysis that shows how risk varies with age, and has been suggested as less sensitive to biases compared to other time-scales and therefore recommended for epidemiological cohort studies [35].

**Fig. 1:**
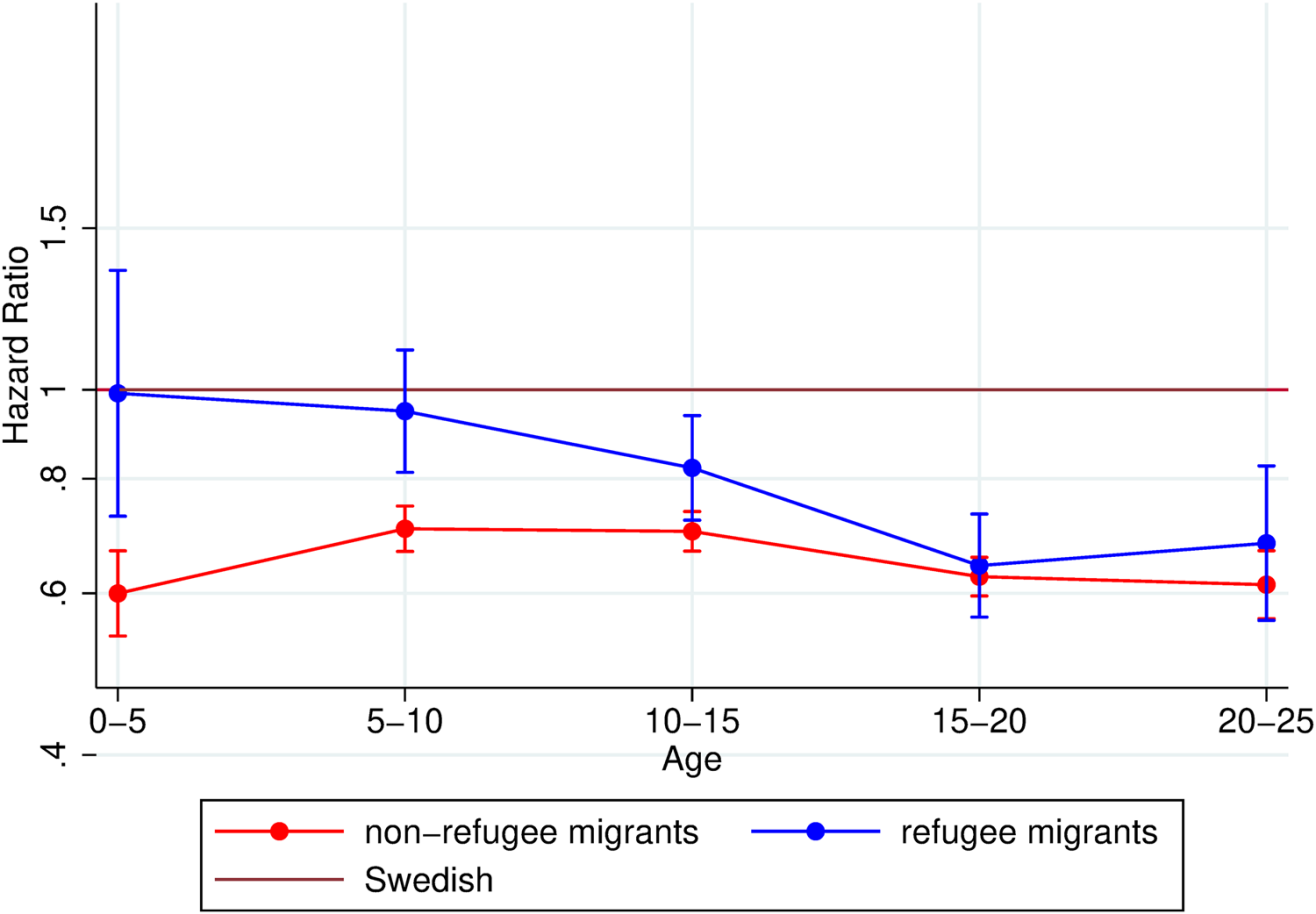
Hazard ratios and 95% confidence intervals of mental health service use among children and young adults comparing migrant sub-groups with Swedish (reference) by age. Adjusted for sex and parental income

**Fig. 2:**
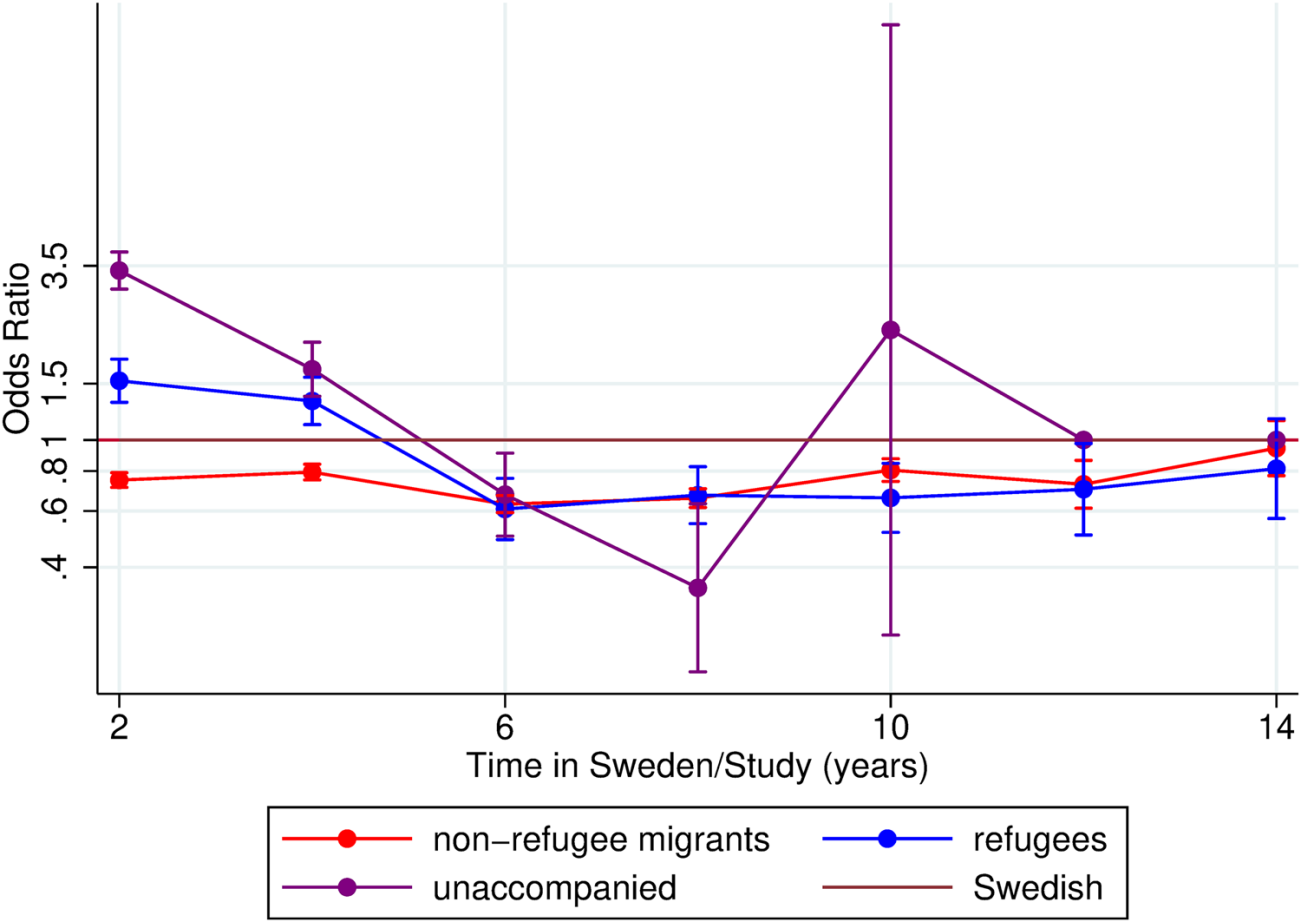
Odds ratios and 95% confidence intervals of mental health service use among children and young adults comparing unaccompanied, accompanied, and non-refugee migrants to Swedish. Adjusted for age and sex

In the first analysis, we estimated hazard ratios for migrants, including refugees but excluding unaccompanied minors, using the Swedish as reference (supplementary figure A). Next, we performed the same analysis but distinguishing refugee migrants and non-refugee migrants, (supplementary figure B), and this analysis was then adjusted for sex and parental income (Fig. 1). The third analysis (Fig. 2) was of the effect of time-in-Sweden on mental health service use. Here, we used logistic regression adjusted for age and sex, to compare migrant sub-groups to all uncensored Swedish individuals at each time-interval throughout the follow-up-period. This was calculated by dividing the follow-up into seven 2-year time-intervals, from 2 to 14 years in Sweden. We first estimated odds ratios comparing refugee migrants and non-refugee migrants to Swedish (supplementary figure C), and then distinguished unaccompanied refugees from accompanied refugees in a more detailed sub-group analysis (Fig. 2).

**Fig. 3:**
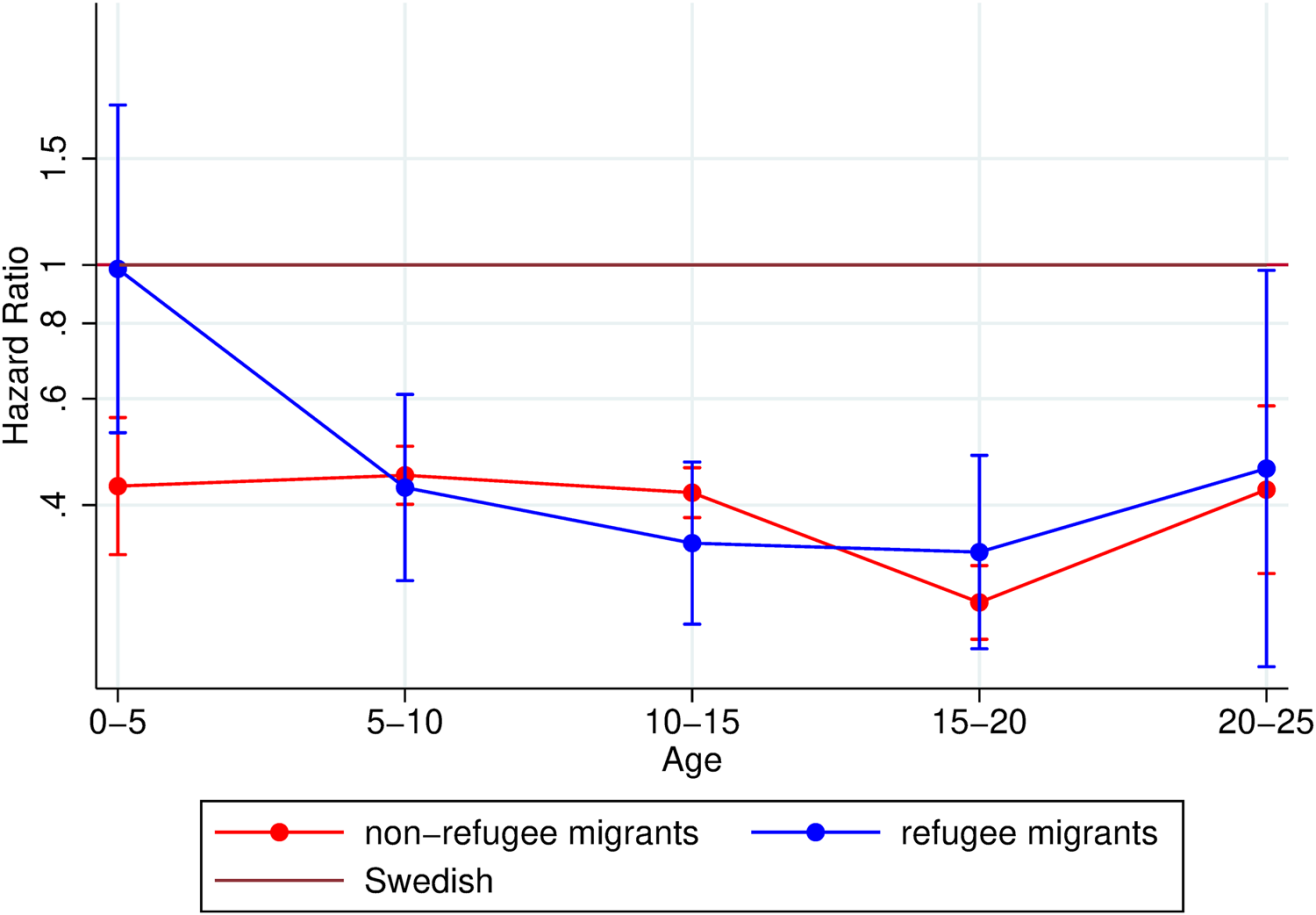
Hazard ratios and 95% confidence intervals of first-time diagnosis of a neurodevelopmental condition among children and young adults comparing migrant sub-groups with Swedish, by age. Adjusted for sex and parental income

Next, we estimated hazard ratios for our secondary outcome of first-time diagnosis with a neurodevelopmental condition. We compared refugee and non-refugee migrants to Swedish in an unadjusted model (supplementary figure D), and subsequently adjusted for sex and parental income in the final model (Fig. 3).

**Fig. 4:**
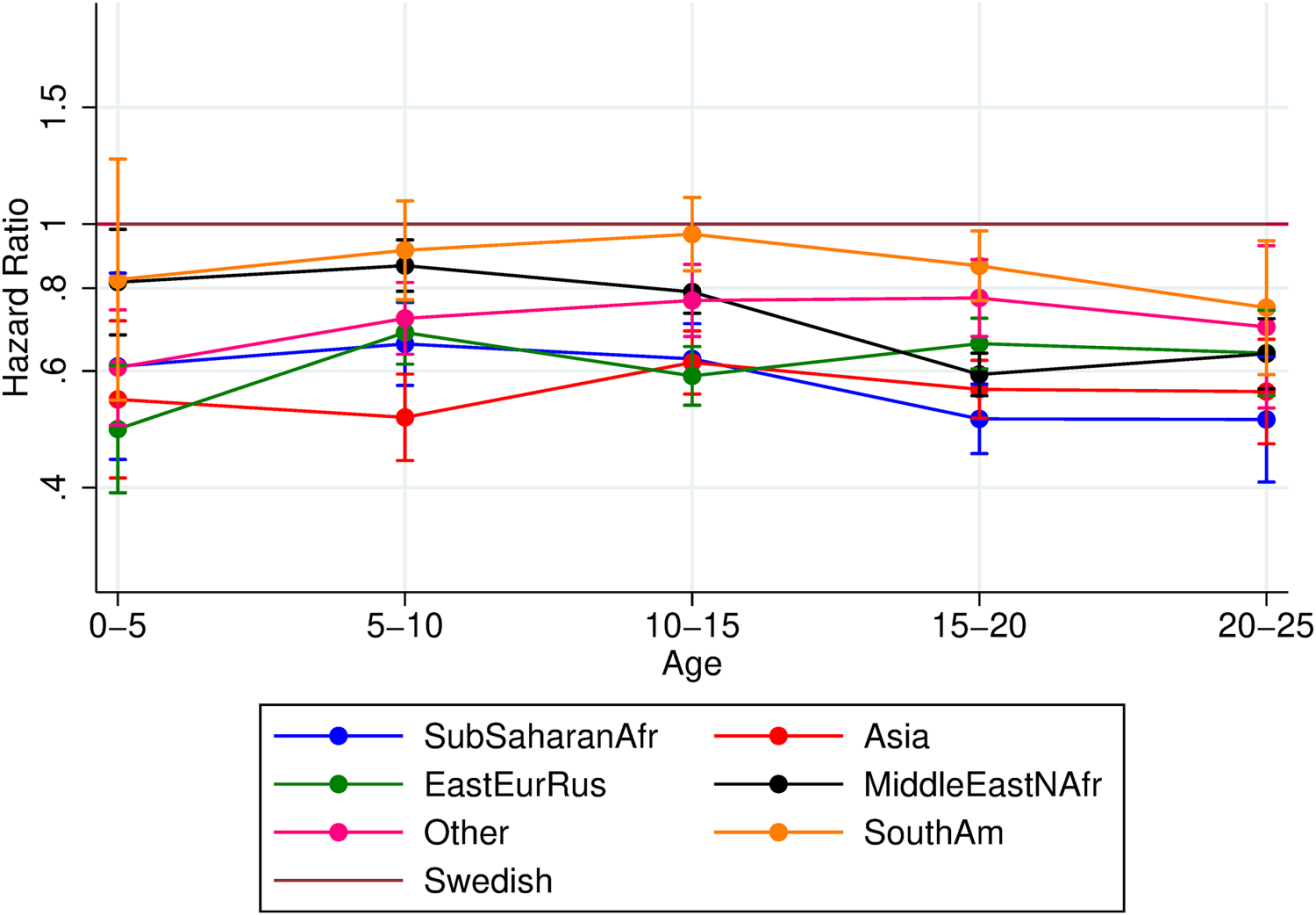
Hazard ratios and 95% confidence intervals of mental health service use among children and young adults comparing migrants from different regions of origin with Swedish, by age. Adjusted for sex and parental income

Finally, to explore the influence of region of origin, we estimated hazard ratios of any mental service use according to region of origin for migrants (Fig. 4).

## Results

The final cohort was made up by 91.40% (*n* = 431,533) Swedish and 8.60% (*n* = 40,612) migrants, see Table 1. Among the migrants, 0.77% were refugees (*n* = 3151 individuals) and 0.27% (*n* = 1277) were unaccompanied. Mean age at start differed significantly between the groups and was 6 years for Swedish and 10 years for migrants. Chi-square tests for income showed significant differences between parents of migrant children and Swedish-born but were non-significant for sex.

**Table 1:** Basic characteristics of migrant children and their Swedish-born comparison group

Migration background	Swedish	Migrants	Total
Frequencies (percent)	431,533 (91.40)	40,612 (8.60)	472,129 (100)
		Non-refugee migrants	
		36,184, (7.66)	
		Refugees (excluding unaccompanied)	
		3151 (0.77)	
		Unaccompanied refugees	
		1277 (0.27)	
Males	219,832 (50.94)	20,838 (51.31)	240,670 (50.97)
Females	211,701 (49.06)	19,774 (48.69)	231,475 (49.03)
Mean age (years)	6.0	10.0	
Region of origin		Africa South of Sahara 5212 (12.83)	(1.10)
		Asia 7075 (17.42)	(1.50)
		Eastern Eur, Russia, Baltics 7805 (19.22)	(1.65)
		Middle East and North Africa 11,637 (28.65)	(2.46)
		Nordics 1884 (4.64)	(0.40)
		South America 2621 (6.45)	(0.56)
		USA, Canada, Oceania 886 (2.18)	(0.19)
		Western and Southern Europe 3422 (8.43)	(0.72)
Total (region of origin)		40,612	(8.60)
Parental income at study start (including individuals with parental income data)			
Lowest income quintile	60,177 (13.94)	30,818 (78.35)	90,995 (19.32)
Lower medium quintile	78,068 (18.09)	3631 (9.23)	81,699 (17.35)
Medium quintile	111,859 (25.92)	2222 (5.65)	114,081 (24.23)
Higher medium quintile	103,114 (23.89)	1225 (3.11)	104,339 (22.16)
Highest income quintile	78,315 (18.15)	1439 (3.65)	79,754 (16.94)
Total (parental income)	431,533	39,335	470,868

In the first analysis, we studied mental health service utilization among migrants using Swedish children as reference, with age-varying estimates (supplementary figure A). Results showed that migrant children utilized less mental health services than Swedish across all ages, and were particularly low among children aged 0–5 and young adults aged 20–25 [HR: 0.62 (95%CI: 0.57; 0.69) and HR: 0.62 (95%CI: 0.57; 0.67), respectively]. The next analysis deepened the results of the previous analysis by separating migrants into refugees and non-refugees (see Fig. 1). We estimated hazard ratios for refugee migrants and non-refugee migrants, as compared with Swedish-born. Adjusting for sex and parental income did not alter the estimates (see supplementary figure B for unadjusted results). Refugee migrants aged 0–5 and 5–10 years had hazard ratios of mental health service use that did not differ significantly from Swedish children, see Fig. 1. From 10 to 15 years and above, refugee children’s hazard ratios dropped and did not differ significantly from non-refugee migrant children’s, whose utilization remained consistently lower than their Swedish counterparts [HR for non-refugee migrants aged 15–20 years: 0.63 (95% CI: 0.60; 066); HR for refugees aged 15–20 years: 0.64 (95% CI: 0.57; 0.73)], see Fig. 1. A sensitivity analysis with migrants with at least one Swedish-born parent as a distinct category showed that this group did not differ from the reference group, except in the youngest age-group, where utilization was lower compared to Swedish (supplementary figure E).

As time in Sweden could be of relevance for mental health service utilization, we tested the effect of this for migrants, using Swedish as reference. We first analyzed refugee migrants and non-refugee migrants compared with Swedish, and found that during the first four years in Sweden, refugee migrants utilized significantly more mental health services than their non-refugee counterparts and Swedish peers [OR during the first 2 years in Sweden for refugee migrants: 2.30 (95% CI: 2.09; 2.54), see supplementary figure C]. In the next analysis, we separated accompanied refugees from unaccompanied refugee minors, to study the effect of reason for migration and parental presence at migration in more detail (Fig. 2). Results showed that the higher odds ratios for mental health service utilization among refugee migrants in their first years in Sweden was largely driven by unaccompanied minors, who had significantly higher odds ratios of utilization during their first 2 years in Sweden compared to Swedish, with an OR of 3.39 (95% CI: 2.96; 3.85). For accompanied refugees, the corresponding OR was 1.53 (95% CI: 1.31; 1.79), see Fig. 2. After approximately 6 years in Sweden, accompanied and unaccompanied refugee children’s odds ratios of utilizing mental health services became significantly lower than Swedish children’s, and after 12 years in Sweden, estimates were no longer significant, see Fig. 2. When analyzing migrants with at least one Swedish-born parent separately, results showed that this group had slightly lower utilization at certain times [OR during the first 2 years in Sweden for migrants with a Swedish-born parent: 0.82 (95% CI: 0.70; 0.96) and OR during the first 6 years: 0.75 (95% CI 0 0.64; 0.86), supplementary figure F.]

To investigate whether the significantly lower utilization of mental health services that we observed for non-refugee migrants of all ages, and for refugee migrants of ages 10–15 years and above (Fig. 1), could be explained by differences in utilization of care for neurodevelopmental disorders, we studied first-time diagnosis of a neurodevelopmental condition for our different exposure groups. Our results showed that, for non-refugee migrants, diagnosis for neurodevelopmental disorder was significantly lower compared to Swedish for all age-groups [HR for non-refugee migrants aged 0–5 years: 0.43 (95% CI: 0.33; 0.56); aged 20–25 years 0.42 (95% CI: 0.31; 0.58] (see Fig. 3). For refugee migrants, hazard ratios were significantly lower than Swedish in all age-groups except for the youngest (aged 0–5 years), where estimates were not significantly different from the Swedish (see Fig. 3). Adjusting for sex and parental income affected estimates only in the oldest age-group, where the divergence from the reference widened (see supplementary figure D for unadjusted results). A sensitivity analysis of migrants with at least one Swedish-born parent showed that this group did not differ from the reference (supplementary figure G).

Finally, we tested the effect of region of origin on mental health service use among migrants. Results showed that migrant individuals from all regions and across all age-groups had significantly lower hazard ratios of mental health service use compared to Swedish, with the exception of individuals from South America, whose utilization did not differ significantly from the Swedish in the ages 0–15 years (see Fig. 4). Adjusting for parental income and gender did not alter the estimates significantly.

### Additional sensitivity analyses

As our definition of the reference category included Swedish-born individuals with one Swedish-born parent, and given that this group’s mental health needs and utilization patterns may diverge from Swedish-born individuals with two Swedish-born parents, we conducted sensitivity analyses excluding this group from the reference category. This did not change our results (data not shown). When analyzing this group separately, we found a slightly higher utilization of overall mental health services compared to Swedish [HR:s ranging from 1.10 (95% CI 1.06; 1.14) to 1.11 (95% CI 1.07; 1.14)]. Utilization for neurodevelopmental disorders did not differ significantly for this group compared to Swedish-born individuals with two Swedish parents in most age-groups, except for those aged 10–20 years, whose utilization was slightly higher than the reference [HR for ages 10–15 years: 1.13 (95% CI 1.06; 1.21) and HR for ages 15–20 years: 1.08 (95% CI 1.03; 1.14)] (supplementary figures H and I).

## Discussion

This study showed that all migrants, irrespective of age, used less mental health services than their Swedish peers, in line with our overall hypothesis. However, among refugee migrants, those who had recently resettled or were below 10 years of age utilized more care or had similar utilization compared to Swedish. The lower utilization of services among the migrant population overall was partially explained by the low utilization of services for neurodevelopmental disorders.

Previous research has shown lower use of psychiatric care among migrant children and adolescents [36–38], and as such, our results corroborate these findings. When we distinguished non-refugee migrants from refugee migrants, the lower utilization patterns observed for the overall migrant population remained unchanged for non-refugee migrants, but only held for refugee migrants above 10 years of age. The youngest refugee migrants had utilization patterns that did not differ significantly from the Swedish. These age-varying effects for refugee migrants have not been previously demonstrated, in large part because the youngest age-groups have not been included in previous register studies [6, 9].

In contrast with a previous Swedish study which found that non-refugee immigrant youth had higher rates of psychiatric care utilization compared to refugees [6], our results indicate the reverse pattern. It is possible that using a more comprehensive outcome measure, which includes primary care as well as specialist pediatric and psychiatric services, better describes the care received by these migrant sub-groups, and that our results more accurately capture differential utilization patterns. Thus, notwithstanding a possibility of lower overall needs among non-refugee migrants, our findings indicate barriers to care.

Our results regarding neurodevelopmental disorders substantiates the previous findings of lower utilization of ADHD-medication among children with an immigrant background [8, 25] and could partially explain the overall lower use of mental health services, evident especially for non-refugee migrants across all age-groups. Although research on the mental health of non-refugee migrants have yielded inconclusive results [7, 39, 40], the previous research suggests that neurodevelopmental disorders are in fact under-diagnosed among migrant children [27]. Consequently, our findings of significantly lower utilization of services for neurodevelopmental conditions can be interpreted both as an indication of under-utilization of care for these conditions, and as a partial explanation for the overall lower use of services observed. While these findings add to our understanding of potential drivers of divergences in service use, more research is needed to clarify the potential role of other mental health conditions, which may also be under-diagnosed.

### Public health implications of this study

Given previous indications of higher risk of poor mental health among refugee youth, our findings of lower mental health service use among adolescent and young adult refugees relative to their younger counterparts and Swedish peers are of noticeable concern. Although it is possible that early detection of mental illness among the youngest refugees reduces the need for care later in life, it is nevertheless possible that adolescent and young adult refugees do not receive appropriate care according to need. While pre-school and elementary school children are regularly examined by child- and school-based health care professionals, adolescents and young adults lack such detection and referral opportunities. Indeed, a Swedish study examining pathways to child and adolescent psychiatric clinics (CAP-clinics) found that children with an immigrant background were more often referred to CAP-clinics by social/legal services, health/mental services, or the school system than children with a Swedish background, and that these associations were stronger the younger the children were at first contact with the CAP-clinics [41]. Our findings suggest that, for refugee children, whose needs have consistently been shown higher than majority peers, referring agents may be vital for initiating adequate contact with mental health services. Collaborations between schools, primary care, social services, and psychiatric services could thus benefit individuals who may otherwise face barriers to reach mental health services.

Accordingly, our finding that unaccompanied minors had higher likelihood of utilization compared to Swedish during their first two years of resettlement indeed indicates that contact with professionals is crucial for refugee children’s use of services. Unaccompanied refugees are provided with a custodian upon arrival and may thus be more readily guided into the health care system. Moreover, established programs to introduce newly arrived refugees into Swedish society are in place during the first 2 years upon receipt of residence permits, and may strengthen the care-seeking process during this time, for both unaccompanied and accompanied refugees. In line with our findings, a recent Swedish register study showed that unaccompanied minors utilized more psychiatric care compared to majority peers (with the exception of ADHD-medication)—suggesting both higher needs and reinforced access for this group [42].

Previous studies have shown that children with parents from low-income countries utilize less psychiatric care compared to children with native born parents [8, 36, 43], and unfamiliarity and lack of experience with psychiatric services in the home country may impede access to care among migrant children and youth even in a setting where services do exist [9]. Notably, our results regarding country of origin showed that specific origins could not explain utilization patterns, in contrast with findings regarding adult migrants (where utilization has been shown particularly low among those from Sub-Saharan Africa, Asia, and Western and Southern Europe) [32]. Our findings could indicate that children and youth’s utilization of mental health services are driven by factors outside the family, such as school/social/health care and legal services, pointing again to the potentially crucial role of such referring and guiding agents. Strengthening the awareness regarding unmet needs, and the referring capacity by professionals working with migrant children and youth could help reduce barriers to care among potentially vulnerable groups.

Besides ensuring equal access to care for those in need, a further step toward greater equality in mental health would be to target some of the root causes of the unequal mental health burden that appear to exist between migrant and majority groups. The literature on the social determinants of health have consistently shown that groups with poorer socio-economic status are at risk of poorer mental health [44], and in the Swedish context, many findings confirm that foreign-born individuals are more often socio-economically disadvantaged compared to Swedish-born individuals [45, 46]. Furthermore, studies indicate that belonging to a visible minority group and experiencing racism is associated with greater risk of mental ill-health among adults [47], and lower self-rated well-being among school children [48]. The interplay between socio-economic disadvantage, structural inequalities and discrimination, and the risk for mental ill-health among vulnerable groups thus deserve political attention and policy intervention aimed at tackling the deeper societal causes of unequal mental health outcomes between migrant and majority populations. Efforts to simultaneously reduce social disadvantage while expanding delivery of care could thus contribute to lessening the disparities in mental health and service utilization [49].

### Strengths and limitations

Using a linked register database, we were able to observe the entire population of children and youth in Stockholm County over a 10-year period, meaning a broad generalizability Stockholm, along with other major cities in Sweden, has a slightly higher immigrant population than the national average. Furthermore, it has a large number of out-patient mental health clinics, reflecting the size of the population living in the Stockholm region (approximately 2.3 million individuals). It is conceivable that urban and rural differences in Sweden in terms of availability of clinics reduces the generalizability of our findings, but on the other hand, Sweden’s universal health care system offers free of cost care to all minors, with the aim of reducing regional disparities. Using only Stockholm data could nonetheless limit the generalizability of our findings to urban settings. However, using Stockholm data meant that we could include primary care and pediatric specialist care for a psychiatric diagnosis, as well as first-time visit to specialist psychiatric services, which allowed for a more comprehensive investigation of utilization. Our results also highlight the usefulness of age-varying analyses to understand differences in utilization among migrant sub-groups, indicating the importance of societal support systems for referral and guidance to mental health care.

We also recognize several limitations in our study. First, for parental socio-economic status, we used income as a proxy. The reason for using income and not education was the higher reliability of income data for migrants; however, a combination measure could possibly be a more accurate way to represent socio-economic position. Second, we did not analyze the potential effect of parental mental illness on the outcome, as data on parental mental health would be greatly limited for migrants, biasing our analyses of these associations. Third, we included both children and young adults in our study, allowing adolescents up to 15 years of age to enter at study start, and older individuals to be included throughout the study period. Our results may thus partly reflect different service provision for children and adults, and it is possible that our observed age differences could partly be explained by differential access and service types for children as compared to youth above the age of 18 years. In addition, for those in compulsory and secondary school, school-based health services are in place and function both as referring and health promoting agents. Thus, school-based mental health services may reach and benefit children and adolescents but were not analyzed here—despite the potentially important mental health care provision of such services. Finally, using a broad outcome definition, we did not distinguish between emergency and non-emergency care, nor between in- and out-patient contacts. An investigation of possible differences in terms of pathways to, and type of care received (i.e., compulsory or voluntary and in- or out-patient), is essential for a more complete understanding of differential access to care. Finally, further investigation of utilization patterns for other mental health conditions (besides neurodevelopmental disorders) is needed to add knowledge to our understanding of differential service use and its probable explanations.

## Conclusion

Overall, this study shows that all migrants 0–25 years of age, irrespective of age, used less mental health services than their Swedish peers. However, refugee migrants below 10 years of age are in contact with services to a greater extent than their older counterparts. Non-refugee migrants utilize less care irrespective of age and time in Sweden compared to their Swedish peers, while unaccompanied utilize more mental health services in their first years in Sweden. Furthermore, the lower utilization of services among the migrant population overall was partially explained by low utilization of services for neurodevelopmental disorders. Given evidence of pervasive mental health problems among refugee children and youth, and indications of higher mental health needs also among non-refugee migrants, our findings suggest inadequate access to mental health services. The relatively higher utilization observed among young refugees and unaccompanied minors early upon resettlement indicates the importance of detection and referring agents outside the family to ensure adequate delivery of mental health services to those most in need.

## Supplementary Information

Below is the link to the electronic supplementary material.

Supplementary file1 (TIFF 267 kb)

Supplementary file2 (TIFF 274 kb)

Supplementary file3 (TIFF 306 kb)

Supplementary file4 (TIFF 282 kb)

Supplementary file5 (TIFF 567 kb)

Supplementary file6 (TIF 573 kb)

Supplementary file7 (TIF 581 kb)

Supplementary file8 (TIF 526 kb)

Supplementary file9 (TIF 557 kb)

## Data Availability

The data used in this study cannot be made publicly available according to Swedish data protection law. Any questions about the data can be addressed to the corresponding author. The statistical code is available from the corresponding author.
