# Supplementary figures and images for: Mental health service use among migrant and Swedish-born children and youth: a register-based cohort study of 472,129 individuals in Stockholm
Source: Soc Psychiatry Psychiatr Epidemiol. 2021 Jul 28;57(1):161–71. doi: 10.1007/s00127-021-02145-2 (PMC8761127; doi:10.1007/s00127-021-02145-2)

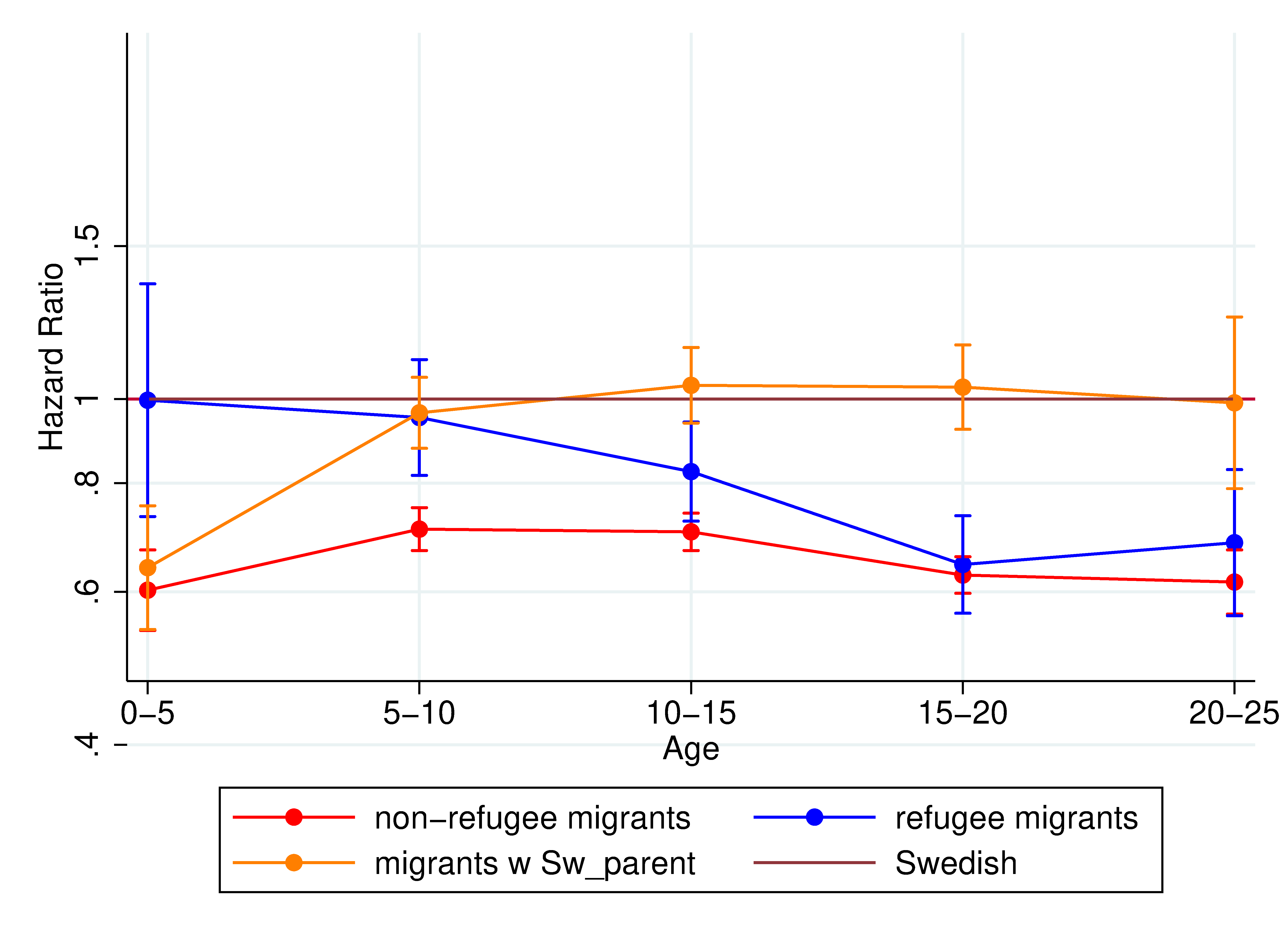

Supplement: Supplementary file 1 — Supplementary file1 (TIFF 267 kb) [file 127_2021_2145_MOESM1_ESM.tiff]

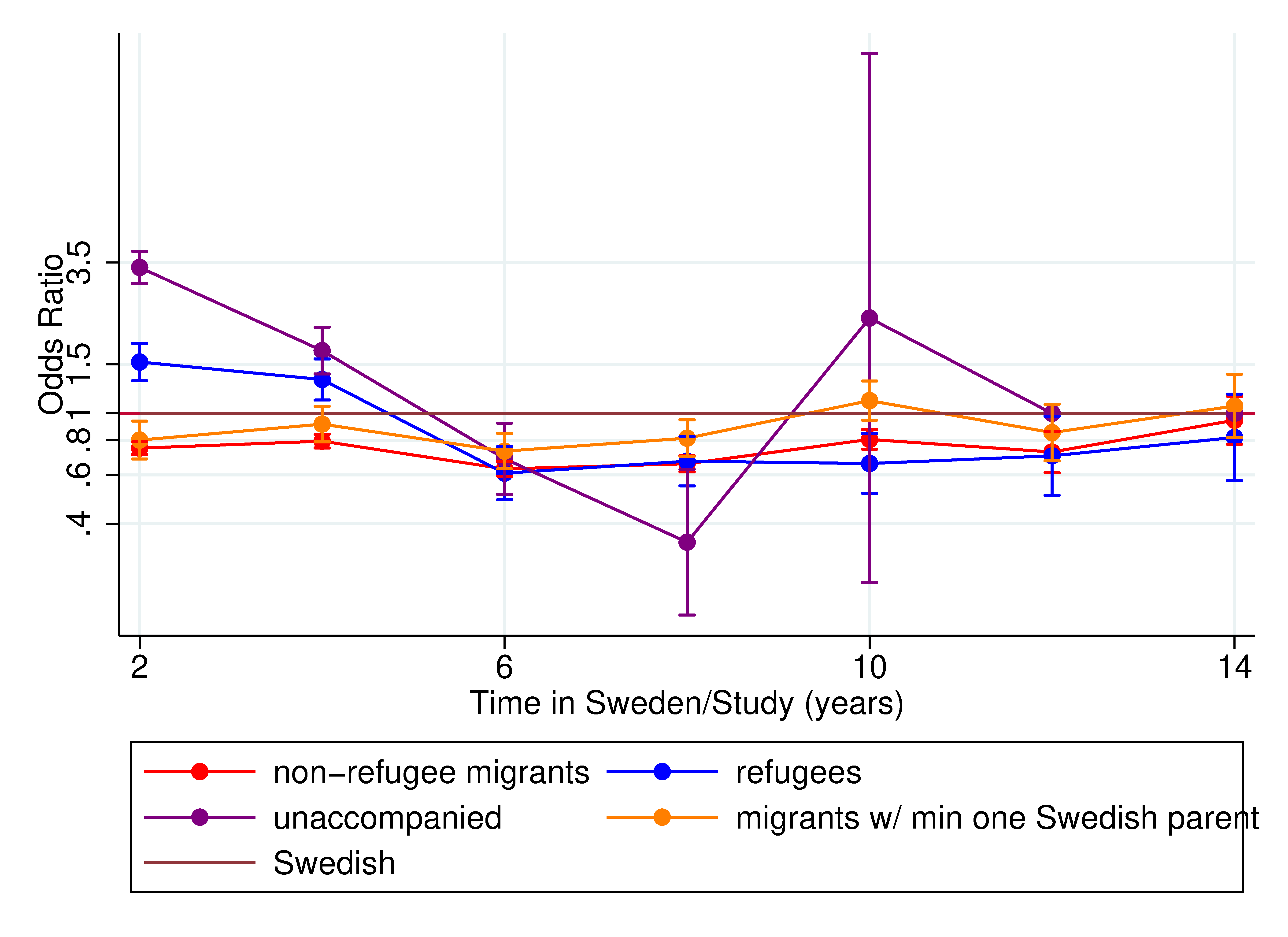

Supplement: Supplementary file 2 — Supplementary file2 (TIFF 274 kb) [file 127_2021_2145_MOESM2_ESM.tiff]

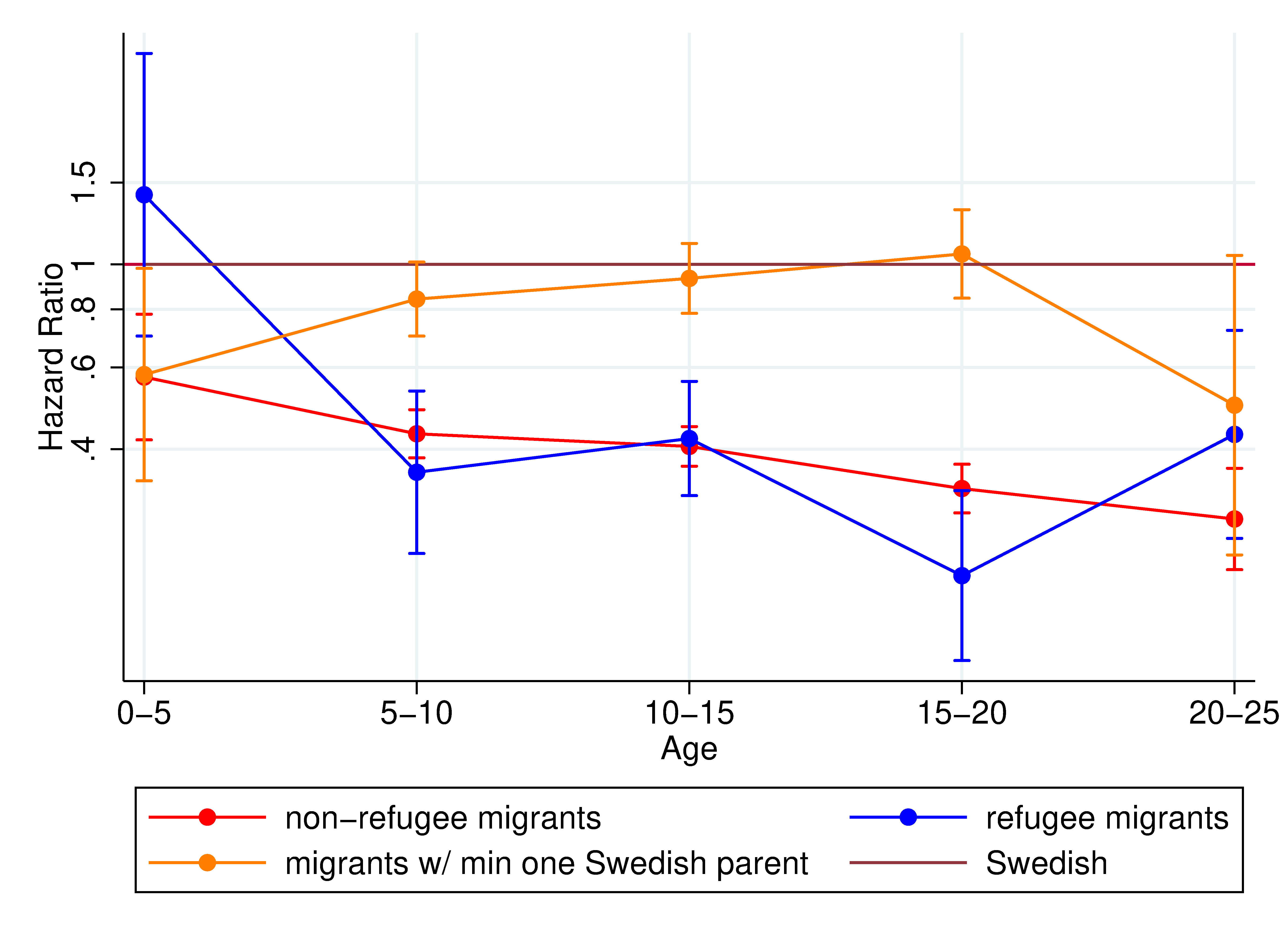

Supplement: Supplementary file 3 — Supplementary file3 (TIFF 306 kb) [file 127_2021_2145_MOESM3_ESM.tiff]

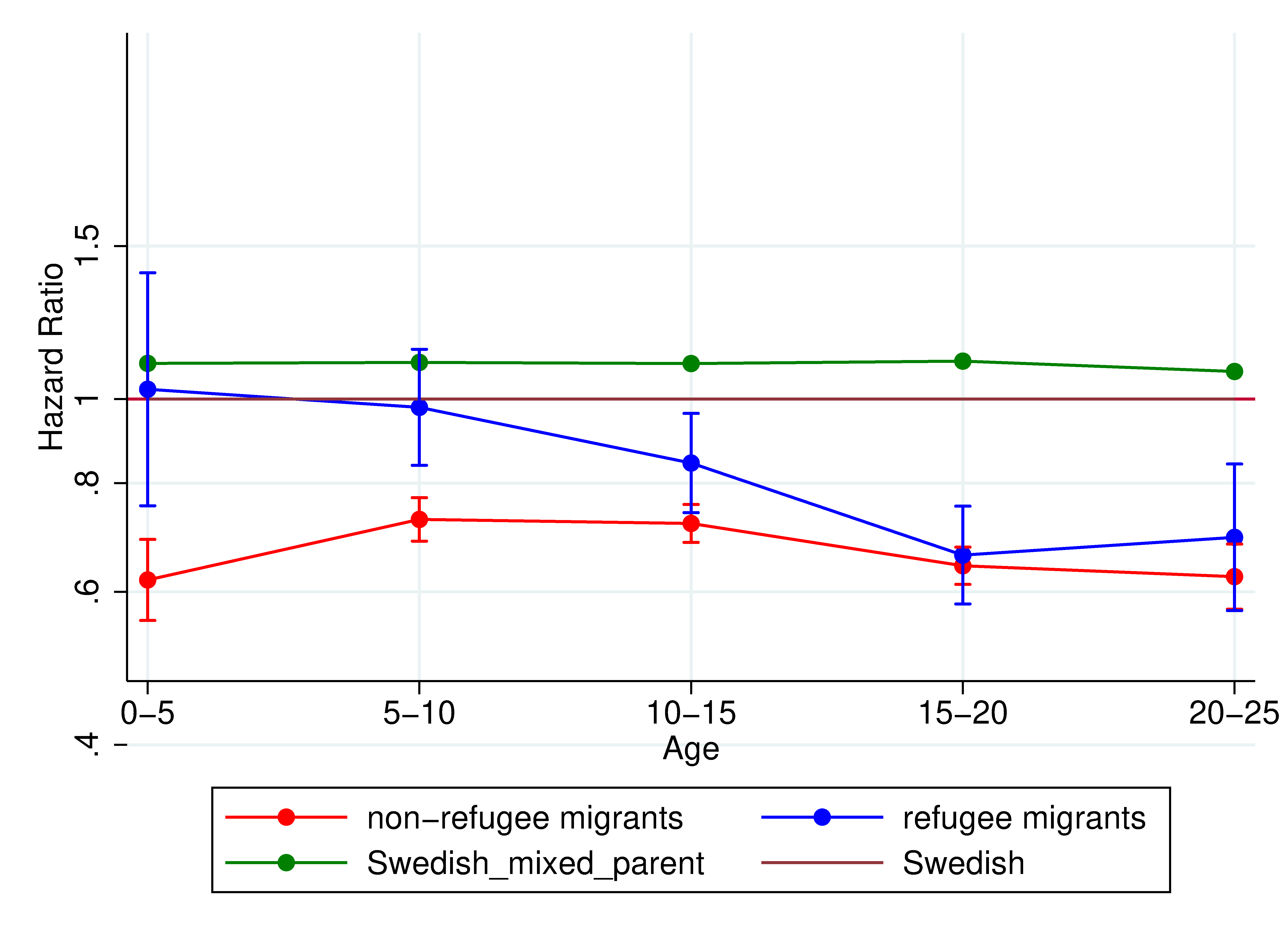

Supplement: Supplementary file 4 — Supplementary file4 (TIFF 282 kb) [file 127_2021_2145_MOESM4_ESM.tiff]

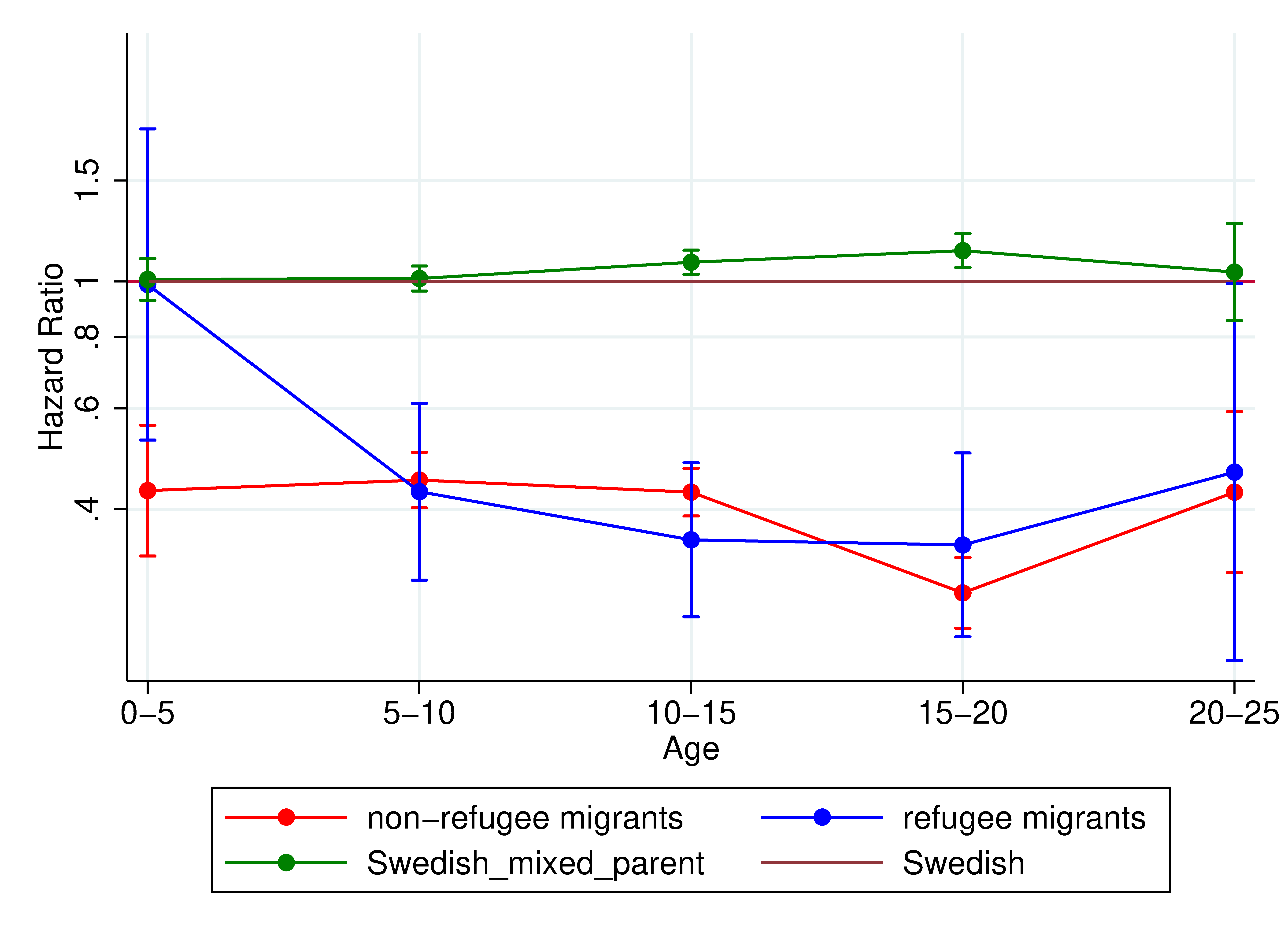

Supplement: Supplementary file 5 — Supplementary file5 (TIFF 567 kb) [file 127_2021_2145_MOESM5_ESM.tiff]

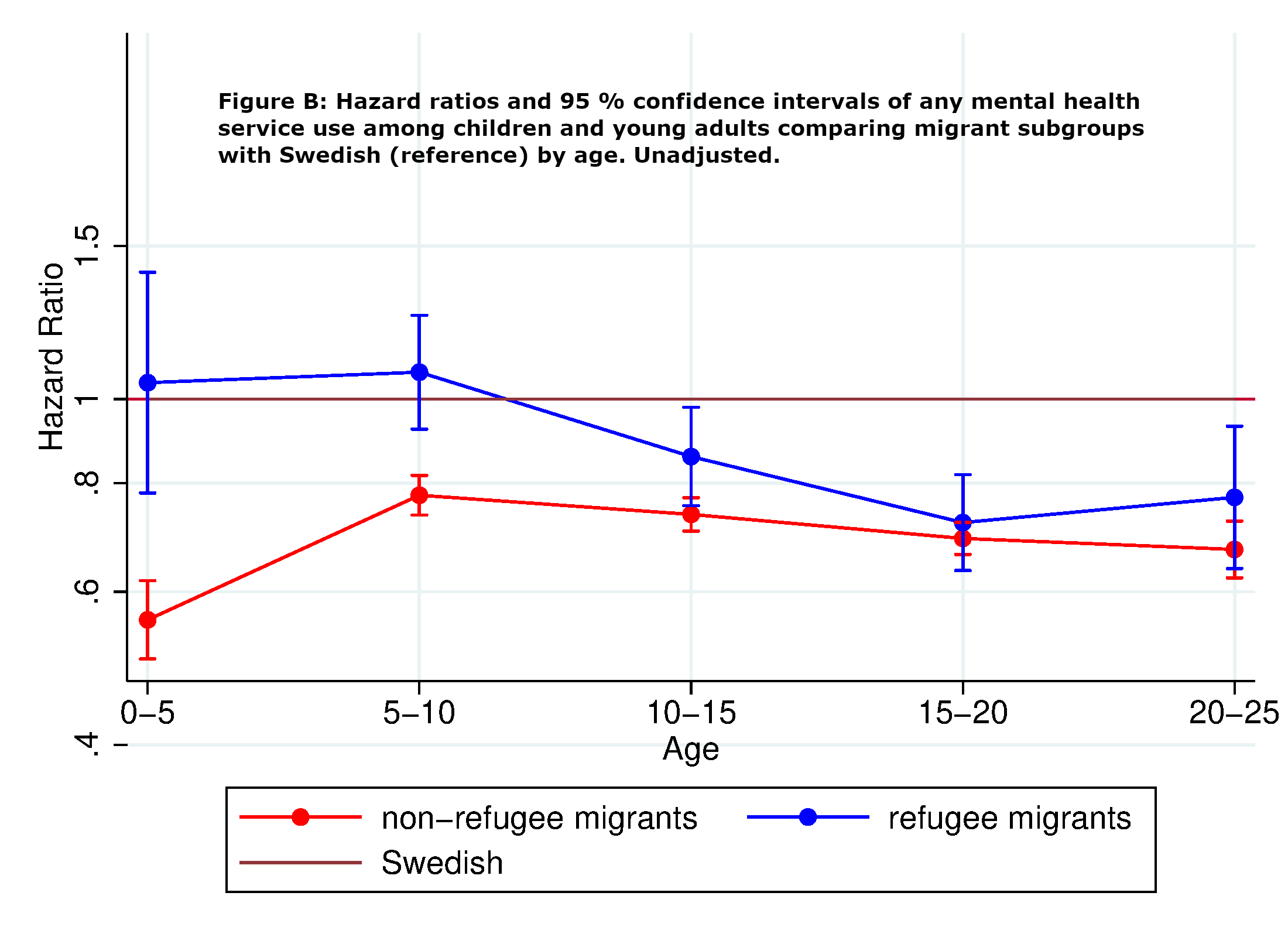

Supplement: Supplementary file 6 — Supplementary file6 (TIF 573 kb) [file 127_2021_2145_MOESM6_ESM.tif]

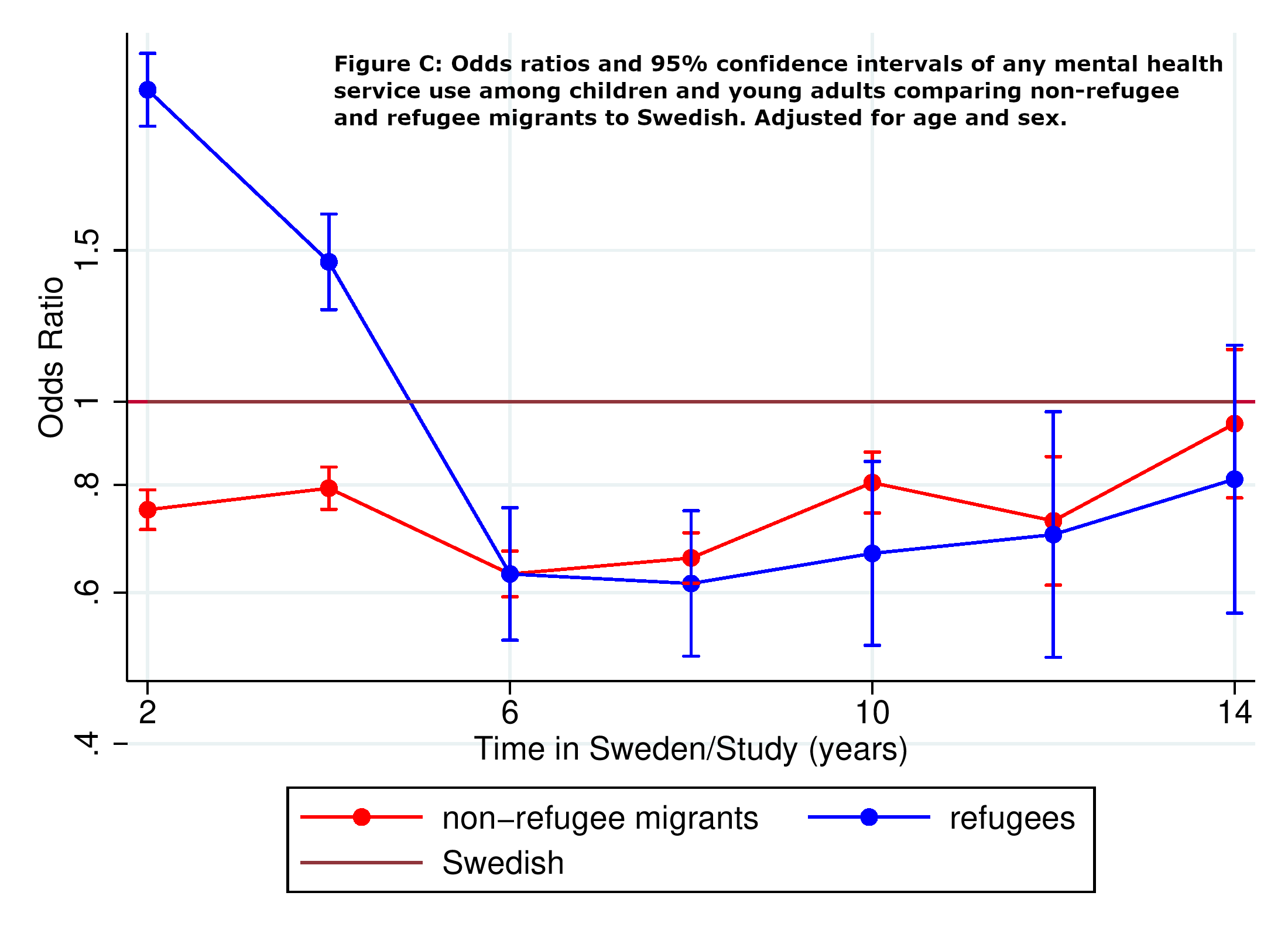

Supplement: Supplementary file 7 — Supplementary file7 (TIF 581 kb) [file 127_2021_2145_MOESM7_ESM.tif]

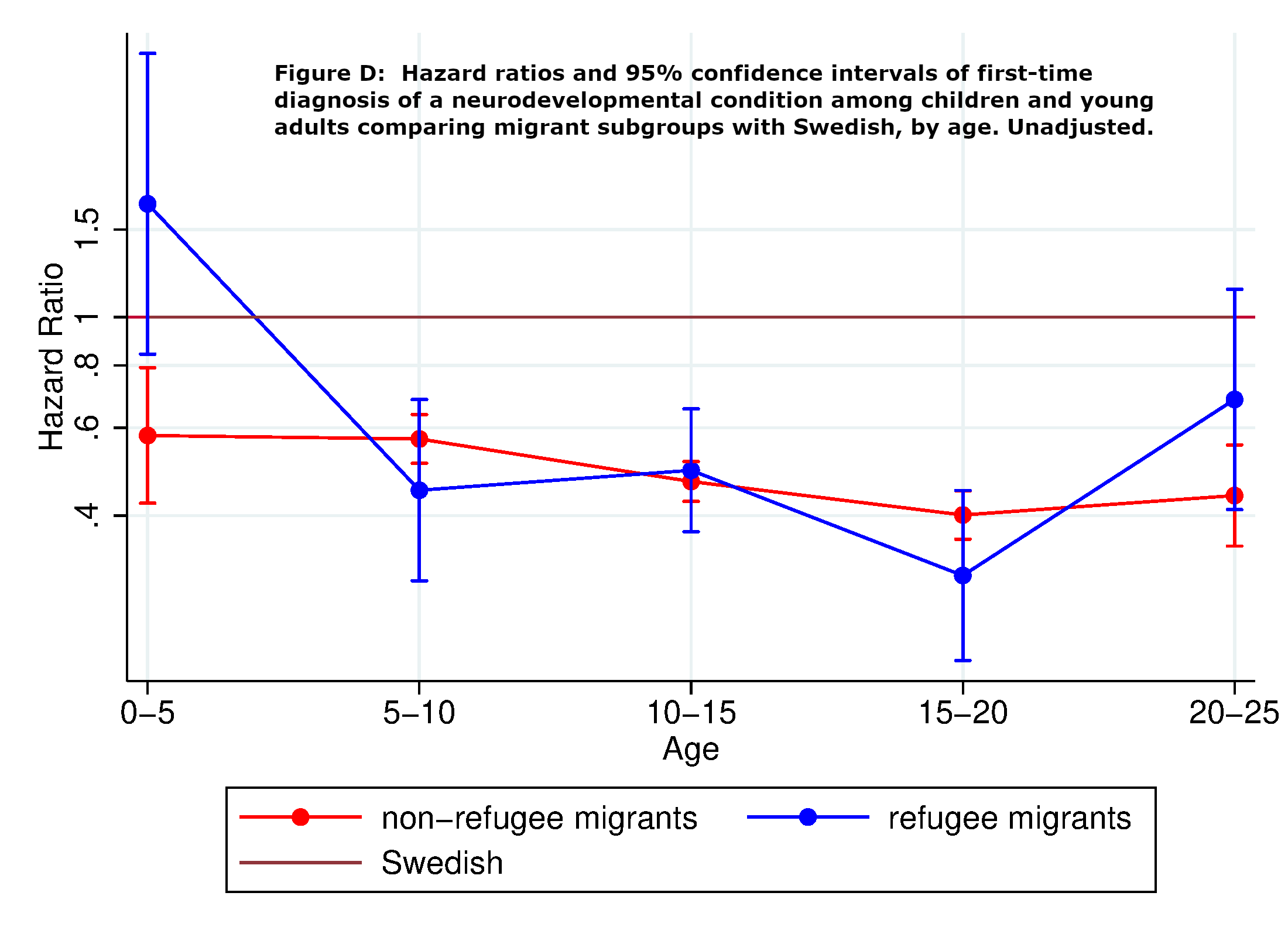

Supplement: Supplementary file 8 — Supplementary file8 (TIF 526 kb) [file 127_2021_2145_MOESM8_ESM.tif]

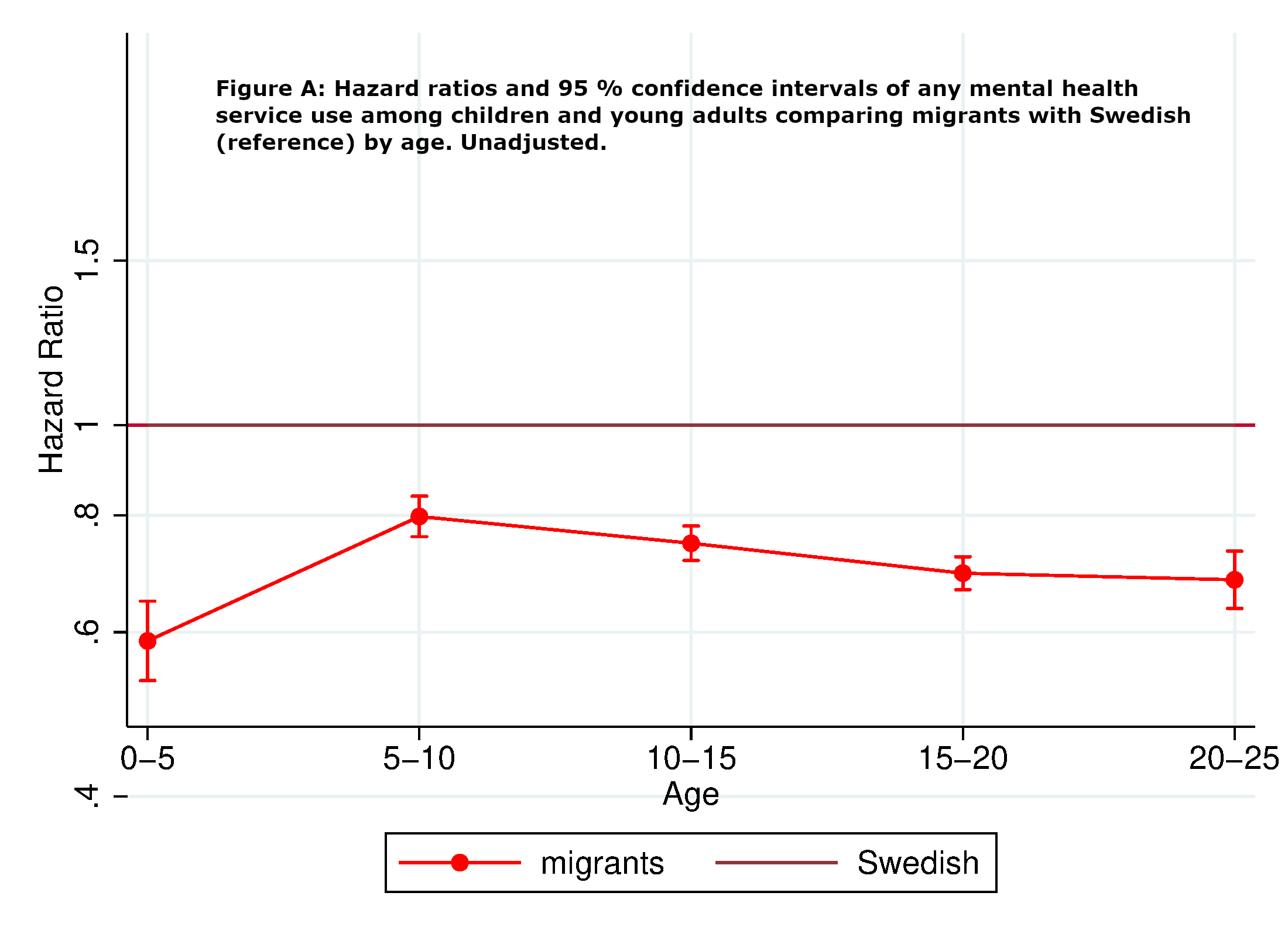

Supplement: Supplementary file 9 — Supplementary file9 (TIF 557 kb) [file 127_2021_2145_MOESM9_ESM.tif]
